# Emerging role of PTEN loss in evasion of the immune response to tumours

**DOI:** 10.1038/s41416-020-0834-6

**Published:** 2020-04-24

**Authors:** Thiago Vidotto, Camila Morais Melo, Erick Castelli, Madhuri Koti, Rodolfo Borges dos Reis, Jeremy A. Squire

**Affiliations:** 1 Department of Genetics, Medicine School of Ribeirão Preto, University of São Paulo, Ribeirão Preto, Brazil; 2 Department of Pathology, Medicine School of Botucatu, Paulista State University, Botucatu, Brazil; 3 Cancer Biology and Genetics, Queen’s Cancer Research Institute, Queen’s University, Kingston, ON Canada; 4 Department of Biomedical and Molecular Sciences, Queen’s University, Kingston, ON Canada; 5 Clinics Hospital of Ribeirão Preto, Ribeirão Preto, Brazil; 6 Department of Pathology and Molecular Medicine, Queen’s University, Kingston, ON Canada

**Keywords:** Cancer genomics, Cancer microenvironment, Prognostic markers, Tumour-suppressor proteins

## Abstract

Mutations in *PTEN* activate the phosphoinositide 3-kinase (PI3K) signalling network, leading to many of the characteristic phenotypic changes of cancer. However, the primary effects of this gene on oncogenesis through control of the PI3K–AKT–mammalian target of rapamycin (mTOR) pathway might not be the only avenue by which PTEN affects tumour progression. PTEN has been shown to regulate the antiviral interferon network and thus alter how cancer cells communicate with and are targeted by immune cells. An active, T cell-infiltrated microenvironment is critical for immunotherapy success, which is also influenced by mutations in DNA damage repair pathways and the overall mutational burden of the tumour. As PTEN has a role in the maintenance of genomic integrity, it is likely that a loss of PTEN affects the immune response at two different levels and might therefore be instrumental in mediating failed responses to immunotherapy. In this review, we summarise findings that demonstrate how the loss of PTEN function elicits specific changes in the immune response in several types of cancer. We also discuss ongoing clinical trials that illustrate the potential utility of PTEN as a predictive biomarker for immune checkpoint blockade therapies.

## Background

Mutations in specific genes, including activating mutations in oncogenes and loss-of-function mutations in tumour-suppressor genes, are critical for cancer development.^1–3^ The inactivation or deletion of tumour-suppressor genes not only facilitates tumour development and progression, thereby keeping these cells in a continuous proliferative state,^4^ but might also influence immune responses.^5,6^ For instance, somatic inactivation or deletion of the gene that encodes the tumour-suppressor phosphatase and tensin homologue (*PTEN*)^7^ leads to the activation of phosphoinositide 3-kinase (PI3K) and subsequent downstream signalling to AKT and mammalian target of rapamycin (mTOR),^8^ a serine/threonine kinase that regulates cell growth, survival and proliferation.^9^ PTEN loss is also linked to aggressive cancer phenotypes.^10^ However, new studies have shown that, in addition to its established role in cancer progression, PTEN deficiency can lead to an immunosuppressive tumour microenvironment (TME)^11–14^ that is unfavourable for effective antitumour immune responses.^15^

Immune and tumour cells often communicate with each other by secretion of factors such as cytokines, chemokines, interleukins and extracellular vesicles.^16^ These signalling molecules can enhance or inhibit the activity of cytolytic cells, primarily represented by natural killer (NK) and CD8^+^ T cells.^17,18^ When CD8^+^ T cells directly recognise tumour antigens as non-self or are presented with tumour antigens through dendritic cells and macrophages, they target and kill the malignant cells.^19^ On the other hand, cytolytic cells are suppressed by cell-to-cell contact with regulatory T (Treg) cells and other immunosuppressive cells, and this is one of the several immune evasion mechanisms that cancers use to suppress antitumour immune responses.^20^

Immunotherapy does not target tumour cells directly: it acts by boosting the natural propensity of the immune system to recognise and respond to the presence of tumours.^21^ The principle of immune checkpoint inhibition involves the use of specific agents that block the suppressive interactions between a developing tumour and the defensive immune system of the patient.^22^ Immune checkpoint proteins such as programmed death ligand 1 (PD-L1), for example, downregulate the immune system and promote self-tolerance by suppressing T cell inflammatory activity against tumours, so that blocking the expression of checkpoint proteins with immune checkpoint inhibitors (ICIs) usually restores the capacity of the immune system to recognise tumour cells and kill them.^23–25^

Therapeutic response of individuals treated with ICIs is highly heterogeneous across tumour types, with some patients showing durable responses in previously incurable and highly aggressive cancers, but the majority of patients still have no appreciable benefit with these new drugs.^26^ Most mechanisms of resistance to ICIs involve functional changes occurring in the TME as well as the acquisition of intrinsic tumour-cell resistance.^27^ Tumour cell-intrinsic somatic mutations function at two different levels: not only do they promote higher levels of cell proliferation and cancer growth but they also activate mechanisms that allow tumours to avoid immune system attack.^28,29^ Recent findings have shown that the overall pattern of somatic mutations and genomic changes in tumours can be associated with resistance to immunotherapy in several different types of cancer,^30,31^ indicating that somatic point mutations, copy number alterations, epigenetic changes and dysregulation of gene expression can profoundly influence the interactions between immune and cancer cells.^29,32^ However, presently, the various molecular determinants of resistance to immunotherapy are complex and poorly characterised.

In this review, we discuss the most recent findings on PTEN in genomic stability, cancer immunogenicity, immune cell infiltration and the immune response across different cancers and summarise the emerging role of PTEN as a predictive biomarker for the use of ICIs. The techniques for detecting PTEN deficiency in tumours are now highly reproducible (see Table 1), which increases the potential utility of this biomarker for disease stratification^33^ and for predicting the chances of a successful response to immunotherapy.

**Table 1:** Laboratory tests for the detection of PTEN loss in tumours.

Technique	Advantages	Disadvantages
Immunohistochemistry (IHC)	Sensitive, rapid and semiquantitative detection. Excellent morphological correlation. Can be used as the main assay to screen for PTEN loss (with FISH as reflex test if required)	Need to use validated method^120^ and an established IHC antibody. Detection of heterogeneity or ambiguous results requires confirmatory FISH of adjacent section
Fluorescence in situ hybridisation (FISH)	Accurate. Good correlation of findings with morphology. Detects genomic heterogeneity and hemizygous deletions	More laborious and costly than IHC. Recommended to perform initial screening by IHC, followed by FISH analysis in cases that are ambiguous or indeterminate by IHC^120^
Quantitative PCR techniques, MLPA	Fast, sensitive detection of clonal gene copy number changes	Cellular or genetic heterogeneity not detected. No morphologic correlation
Sequencing-based gene dosage analysis and detection of point mutation	Highly sensitive for detection of somatic point mutations, partial deletions and indels	Large PTEN deletions and copy number heterogeneity not easily detected

## The functions of PTEN

*PTEN*, one of the most commonly somatically mutated or deleted genes in cancer,^7^ is located at the 10q23.31 cytoband and consists of 9 exons that encode the full-length molecule of 403 amino acids.^34,35^

### Cytoplasmic functions of PTEN

In the cytoplasm, the PTEN protein acts as a dual-specificity phosphatase and a direct antagonist of PI3K signalling by converting the second messenger phosphoinositol-(3,4,5)-trisphosphate into phosphoinositol-(4,5)-bisphosphate, which inhibits downstream signalling pathways^36^ that would otherwise normally mediate downstream activation of the AKT protein. Phospho-AKT activates different substrates, such as mTOR,^37^ a serine/threonine kinase that regulates cell growth, survival and proliferation. Somatic *PTEN* inactivation in tumours causes total loss of PTEN function that disrupts not only its catalytic phosphatase activities but also its regulatory control of the PI3K pathways. PTEN loss leads to downstream changes that govern important cellular processes crucial to cancer progression, including survival, proliferation, energy metabolism and changes in cellular architecture.

### Nuclear functions of PTEN

In addition to its cytoplasmic functions in regulating cell growth and proliferation, PTEN regulates genome integrity and the stability of DNA repair in the nucleus.^38^ *Pten*-null mice show increased genomic and chromosomal instability, leading to centromere disruption, chromosomal translocations and spontaneous DNA double-stranded breaks that appear to occur independently of the PI3K–AKT–mTOR pathway. The protective role of PTEN in the genome is also supported by the finding that the *PTEN*.R189X mutation (which lacks the C-terminal region that is responsible for binding to the centromere protein CENP-C) leads to a significant increase in chromosomal aberrations, with transfected *PTEN*189 cells developing high levels of aneuploidy. This mechanism of protection is directly regulated by the interaction of PTEN with CENP-C, as well as by PTEN regulating the expression of Rad51 and its influence on the double-stranded break repair machinery.^39^ In glioblastoma, DNA repair is attenuated after cell exposure to ionising irradiation when nuclear PTEN is phosphorylated at position 240.^40^ The phosphorylated PTEN binds to chromatin and recruits RAD51 to promote DNA repair. In prostate cancer, PTEN also binds and promotes the degradation of the DNA-binding factor chromodomain helicase DNA-binding protein 1 (CHD1); PTEN deficiency leads to CHD1 protein stabilisation, which then engages the H3K4me3 epigenetic mark to activate transcription of downstream pro-tumorigenic and pro-inflammatory tumour necrosis factor α (TNFα)/nuclear factor κB (NF-κB) gene networks.^41^ NF-κB is a complex transcription system governing a diverse set of response genes mediating inflammatory and stress responses. The effects of PTEN on NF-κB transcription are also regulated by a translational variant of PTEN, called PTEN-L. The specific effects of loss of PTEN and PTEN-L on NF-κB-related immune responses are discussed below.

PTEN also regulates cellular senescence (i.e. irreversible arrest of cellular proliferation) mechanisms in cells that have lost proliferative capacity (reviewed in ref. ^42^). Loss of PTEN induces cellular senescence as a failsafe mechanism to defend against tumorigenesis.^43^ In prostate cancer, *PTEN* loss has been shown to activate p53-dependent cell senescence by mTOR kinase binding to p53.^43,44^ Concomitant loss of p53 allows cells to override the cytostatic effects of PTEN-induced senescence. Nuclear PTEN also interacts with the anaphase-promoting complex (APC) and regulates cellular senescence through APC–cadherin 1-mediated protein degradation.^45^ Moreover, senescence activation in PTEN-deficient cells is also associated with cytokine secretion that leads to an immunosuppressive TME.^46^

**Fig. 1:**
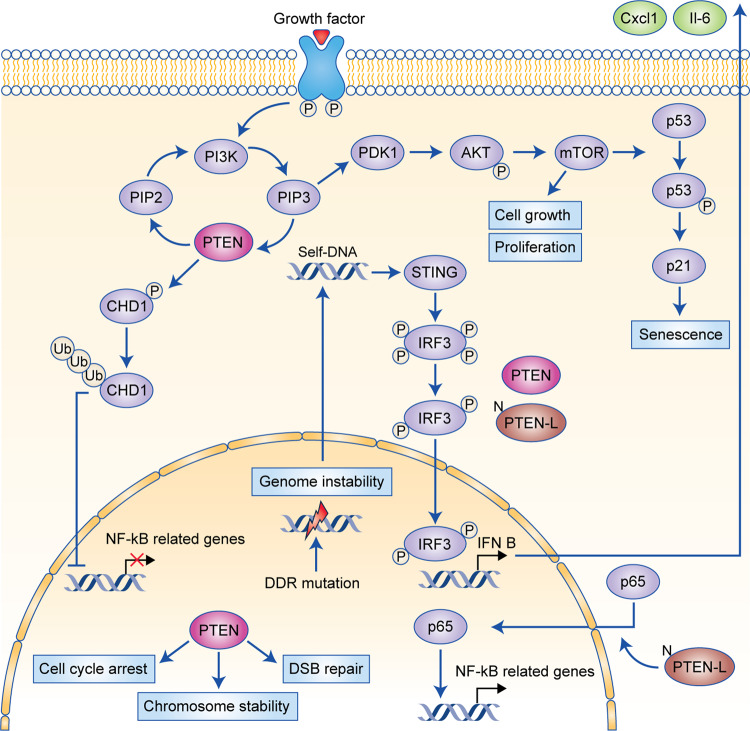
PTEN functions in the cytoplasm and nucleus of cells. Tumour-suppressor functions: The PI3K–AKT–mTOR pathway is negatively regulated by PTEN in the cytoplasm through the dephosphorylation of phosphatidylinositol (3,4,5)-trisphosphate (PIP_3_) to phosphatidylinositol (4,5)-bisphosphate (PIP_2_). Increased activation of PI3K–AKT–mTOR leads to abnormal cell growth and proliferation. Cell senescence: PTEN also regulates cell senescence through the PI3K–AKT–mTOR network: the mTOR complex directly phosphorylates p53 that promotes the accumulation of p21. Consequently, p21 induces cell senescence. Nuclear PTEN also interacts with the anaphase-promoting complex (APC) and regulates cellular senescence through an APC–cadherin 1 complex. In this manner, PTEN loss promotes cell senescence as a failsafe against tumorigenesis. Immune and inflammatory response: PTEN negatively regulates the nuclear factor κB (NF-κB) signalling pathway through chromodomain helicase DNA-binding protein 1 (CHD1), which is ubiquitinated (Ub) in the presence of PTEN and thus is unable to promote the transcription of NF-κB genes in the nucleus. On the other hand, PTEN-L promotes the nuclear import of p65, which consequently induces the transcription of NF-κB genes. The presence of cytoplasmic DNA—as a consequence of genomic instability—activates the STING pathway, which phosphorylates interferon-regulatory factor 3 (IRF3). PTEN and PTEN-L are required for the migration of IRF3 into the nucleus, where this transcription factor mediates the immune response by promoting the expression of type I interferon (IFN) genes, such as interleukin (IL)-6 and chemokine (C-X-C motif) ligand 1 (CXCL1). DNA integrity: The concomitant presence of DNA damage repair (DDR) gene mutations or genome instability leads to double-stranded breaks (DSBs) in the DNA, which often causes self-DNA to migrate into the cytoplasm. Such genomic changes are also controlled by PTEN, since this tumour suppressor regulates cell cycle checkpoints, maintains centrosome stability and is involved in DNA repair.

The various established PTEN signalling pathways associated with PI3K, genome stability and emerging features related to immune responses are summarised in Fig. 1.

### *PTEN* inactivation, genomic instability and cancer immunogenicity

**Fig. 2:**
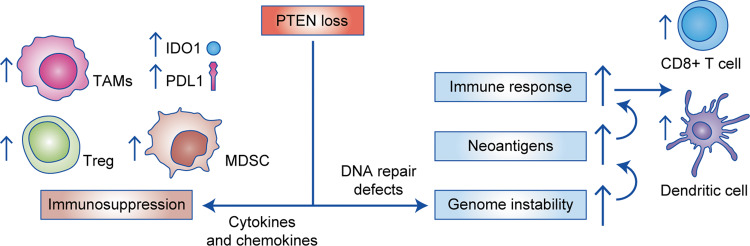
Pro- and anti-inflammatory effects of PTEN deficiency on the immune response of cancer. PTEN loss is associated with cytokine and chemokine signalling that creates an immunosuppressive microenvironment. The TME becomes populated with immune cells that suppress the antitumour response, such as myeloid-derived suppressor cells (MDSCs), regulatory (Treg) cells and M2 macrophages (left side). PTEN-deficient tumours have also been linked with higher indoleamine 2,3-dioxygenase 1 (IDO1) and PD-L1 expression, which are known to reduce the activity of cytotoxic immune cells capable of killing cancer. In contrast, PTEN deficiency may also result in pro-inflammatory effects due to loss of the various nuclear functions of PTEN (right side). For example, there are higher levels of genomic instability, which may result in tumours that produce neoantigens. Neoantigens can elicit an immune response and activate CD8^+^ T cells. However, to counteract the effects of neoantigens, it is likely that tumours with high levels of genomic instability are able to suppress host immune responses to counter pro-inflammatory activity. TAM tumour-infiltrating macrophage.

As outlined above, PTEN normally regulates several mechanisms that maintain genome stability in the nucleus, so PTEN-deficient cancers are known to exhibit high rates of chromosome rearrangements^47,48^ that are usually associated with increased mutational load.^49^ To better understand the consequences of aneuploidy in tumours, >10,000 tumours from The Cancer Genome Atlas were investigated in two studies.^49,50^ Both reports found that high levels of aneuploidy (defined as a whole chromosome or chromosome arm imbalances) were associated with decreased immune response levels, as determined by a low level of lymphocyte infiltration^49^ and a high expression of anti-inflammatory cytokines.^50^ However, this observation—that tumours with high aneuploidy levels are infiltrated by leukocytes to a lesser extent—seems to contradict the concept that increased levels of genomic change (higher mutational load) are often associated with increased immunogenicity.^32^ Tumours with increased mutational burden are more likely to produce neoantigens, which can be recognised as non-self and elicit an immune response. Increased immunogenicity may lead to a higher immune-cell abundance in the TME, improved patient survival and a better response to ICIs.^51,52^ As *PTEN* inactivation promotes higher rates of genomic instability,^47^ it would be generally expected that PTEN-deficient tumours would be pro-inflammatory, having a greater mutational burden and exhibiting increased immunogenicity in the TME. However, for many of the aneuploid tumours, it seems that immunoresistance may be able to suppress the inflammatory antigenic effects of higher mutational loads and increased genomic change^49^ (see Fig. 2).

**Fig. 3:**
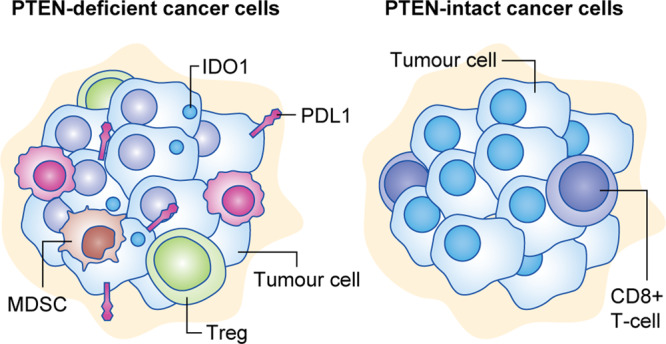
Proposed model of the TME of PTEN-deficient and PTEN-intact cancer. Tumour cells harbouring PTEN loss are linked to a highly immunosuppressive environment mediated by myeloid-derived suppressor cells (MDSCs), regulatory (Treg) cells and M2 macrophages. In this model, the various changes caused by PTEN loss (shown schematically in Fig. 2) are thought to interact to suppress antitumour responses. There are likely to also be uncharacterised tumour-specific differences in immune response. For example, there is no consensus for the relationship between PTEN loss and CD8^+^ T cell density (for more details, see Table 2). TAM tumour-infiltrating macrophage, PD-L1 programmed death-ligand 1, IDO1 indoleamine-pyrrole 2,3-dioxygenase.

Emerging evidence indicates that, in addition to growth-promoting PI3K alterations, PTEN loss is also associated with an immunosuppressive tumour state^11,47,53–55^ that favours cancer progression.^53,54,56,57^ For instance, in a murine model of T cell acute lymphoblastic leukaemia, *Pten* deficiency was reported to promote cell survival in a previously unsupportive TME.^58^ As discussed above, PTEN loss triggers senescence as a failsafe protection mechanism against cancer onset. Depending on the genetic changes already present in the tumour, PTEN-induced senescence can elicit changes in the cytokine network that result in an antitumour immune response in the TME^46^ (see Figs. 2 and 3).

## PTEN and the immune response

Several reports have shown that mutated oncogenes and tumour-suppressor genes have secondary functions in regulating the immune response in the TME in addition to their primary role in cell proliferation and survival in cancer (reviewed in ref. ^5^). In addition to its tumour-suppressor functions, PTEN normally influences the innate immune response by dephosphorylating Ser^59^ of interferon regulatory transcription factor 3 (IRF3) to enable its nuclear migration.^13^ Once in the nucleus, IRF3 promotes the transcription of type I interferon (IFN)-response genes.^13^ Type I IFN responses promote the expression of several genes that (i) control the response against infectious agents, (ii) modulate antigen presentation and cytokine expression and (iii) activate the T cell- and B cell-mediated immune response.^60^ Therefore, the activation of the type I IFN network is key in balancing the adaptive and innate immune responses. Both PTEN and PTEN-L have been shown to influence the transcription of type I IFN-regulated genes during viral infections by regulating IRF3 activation. PTEN-L regulates the import of IRF3 and the NF-κB component p65 into the nucleus, where both transcription factors activate type I IFN response and NF-κB-regulated genes, respectively.^14^ NF-κB regulates both immune and inflammatory responses and tumour-cell proliferation and apoptosis.^61^ PTEN-mediated enhancement of the type I IFN response is expected to promote the recruitment and activation of antitumour T cells and NK cells in addition to stimulating antigen presentation by dendritic cells.^62^ It is known that the hyperactivation of type I IFN responses is strongly linked to a better response to immunotherapy and increased activation of antigen presentation by tumour cells (reviewed in ref. ^63^). As a result, PTEN-deficient tumours might show impaired activation of both the type I IFN and the NF-κB pathways, which could be highly favourable for tumour progression because of an immunosuppressed TME.

The host antitumour immune response can also be triggered by cancer-specific genomic alterations and mutations.^32^ For instance, as a consequence of double-stranded DNA breaks caused by the presence of inactivating mutations in DNA damage repair (DDR) genes, there might be increased levels of DNA fragments in the cytoplasm of tumour cells.^64^ These increased levels of DNA fragments might activate the cytosolic double-stranded DNA-sensing cGAS-STING pathway, which promotes the phosphorylation of IRF3 (see Fig. 1), thereby promoting its retention in the cytoplasm. In this manner, PTEN deficiency in combination with DDR-mutated tumours might further attenuate the levels of immune response activation due to the lack of transcription of IFN-regulated genes by IRF3.^65^ Cao and colleagues^14^ showed that cells expressing only PTEN-L synthesised the highest levels of C-X-C motif chemokine ligand (CXCL) 10 and the pro-inflammatory cytokines interleukin (IL)-6 and CXCL1. In addition, Wang and colleagues^66^ demonstrated that the knockdown of *PTEN*-*L* significantly reduced the expression of TNFα and IL-6, which both have a pro-inflammatory role in mediating immune response. In this context, loss of PTEN and its secreted variant PTEN-L in tumour cells is likely to increase the activity of immunosuppressive cytokines on the immune cells in the TME. Interestingly, another three translational variants of PTEN exist; however, little is known about their effects in the immune response (reviewed in ref. ^67^). Thus the emerging role of PTEN in mediating the immune response is complex and involves multiple pathways that collectively repress antitumour responses in the TME (shown schematically in Figs. 2 and 3).

PTEN is also known to play a crucial role in the regulation of signalling in immune cells.^12^ *Pten* deletion of specific immune cell subsets in mice cause defects in T cells,^68^ Treg cells^69^ and B cells.^70^ Myeloid cell-specific PTEN deficiency and increased PI3K levels reduce inflammation, increase macrophage phagocytic ability and facilitate resistance to infection.^71^ These findings are consistent with the early observation that PTEN deficiency was associated with chronic inflammation and autoimmunity.^72^ Since inflammation and immunity are known key factors in tumour progression,^73^ it is important to understand how PTEN mediates responses in the TME.

### PTEN deficiency and the immune response in cancer

The impact of PTEN loss and PI3K activity on T cell-mediated antitumour responses was investigated in a preclinical model of melanoma. Inhibition of *PTEN*-null tumours with a PI3K inhibitor led to in vitro killing by T cells and restoration of antitumour control of immune checkpoint inhibition using anti-PD-L1 and anti-cytotoxic T-lymphocyte-associated protein 4 (anti-CTLA4)^30^ antibodies. However, the authors demonstrated that PD-L1 and major histocompatibility complex class I expression do not constitute the primary mechanism of immunosuppression mediated by PTEN loss in melanoma. Their xenografting experiments suggested that the lack of response to ICIs could have resulted from a higher expression of vascular endothelial growth factor (VEGF) and increased secretion of inhibitory cytokines by tumour cells lacking PTEN.

PTEN loss was also found to be significantly associated with altered macrophage densities and CXCL8 expression in a study conducted using 28 radical prostatectomy-derived specimens.^74^ CXCL8 is a pro-inflammatory chemokine that binds to the C-X-C chemokine receptor type 1 CXCR1 and CXCR2. Together, the CXCL8–CXCR1/2 axis mediates tumour initiation and progression, in addition to promoting neovascularisation of the TME.^75^ Haploinsufficiency of *Pten* combined with oncogenic *Kras* mutations was associated with NF-κB pathway activation in a mouse model of pancreatic ductal adenocarcinoma. The authors of this study also showed that stimulation of the NF-κB network led to TME remodelling through the infiltration of immune cells with pro-tumorigenic properties.^76^

Immune checkpoint inhibition is most often met with little or no success in prostate cancer, owing to low levels of immune-cell infiltration. In *Pten*-null prostate conditional mice, tumours are strongly immunosuppressed as a result of the downregulation of the tyrosine-protein phosphatase PTPN11 and consequent Janus kinase 2 (Jak2)–signal transducer and activator of transcription 3 (Stat3) pathway activation.^46^ In another study conducted with *Pten*-deficient mice, additional ablation of the growth-regulatory kinase extracellular signal-regulated kinase 5 (ERK5) promoted T cell infiltration into prostate tumours through Ccl5 and Cxcl10 production.^77^ Thus the concomitant loss of *ERK5* was able to restore immune functions caused by PTEN deficiency leading to tumours with higher T cell infiltration. These data provide important clues about why human prostate cancer does not always benefit from current immunotherapeutic approaches.

PTEN loss has also been linked to changes in the secretome (that is, cell-secreted molecules, such as chemokines and ILs) so that its role in mediating immune responses in the TME might also be indirect. In non-small cell lung cancer, CXCR4 expression was shown to be dependent on the PI3K–AKT–mTOR signalling pathway, which is directly controlled by PTEN.^78^ The loss of Pten in stromal fibroblasts of mouse mammary glands led to the altered production of cytokines and chemokines in the TME, increased macrophage recruitment and enhanced vascularisation, which together accelerated the initiation, progression and malignant transformation of mammary epithelial tumours.^79^ Another study conducted with murine prostate epithelial cells and human prostate cancer cell lines demonstrated that the loss of PTEN enhanced the expression of CXCR4 and CXCL12.^80^ Moreover, in prostate cancer cell lines and prostatic tissue, Maxwell and colleagues^81^ demonstrated that the expression and secretion of the pro-inflammatory chemokine CXCL8 were also associated with PTEN inactivation.

A recently published study conducted in mice demonstrated that the diversity of genomic changes in prostate cancer can determine the composition of immune cells within the TME.^82^ The authors showed that tumours derived from *Trp53*^−/−^*Pten*^−/−^ tumours expressed high levels of Cxcl17, which recruited cells with immunophenotypes of monocytic myeloid-derived suppressor cells (MDSCs) and monocytes. Moreover, these tumours exhibited a Treg cell-mediated immunosuppressive signature that was linked to the tumour-promoting effect of MDSCs. The details of the role of MDSCs in PTEN-deficient tumours are discussed below. On the other hand, simultaneous ablation of *Pten* and *Pml* in mice was related to an ‘immune desert’ phenotype, characterised by low T cell densities and immune checkpoint expression. In summary, changes in *Pten* and the interplay with other genes studied in mouse models of prostate cancer have been shown to collectively shape the immune-cell content and expression of immunosuppressive markers in the TME.

*PTEN* also undergoes germline-inactivating mutations that are responsible for various human syndromes.^83^ The PTEN hamartoma tumour syndrome is a hereditary tumour syndrome that predisposes patients to benign and malignant breast, thyroid, renal cell and endometrial tumours.^84^ A recently published study demonstrated that the innate immune cells from patients with PTEN hamartoma tumour syndrome produce high levels of lactate.^85^ Lactate confers a pro-inflammatory phenotype of monocytes in the TME of PTEN-deficient thyroid cancer. Interestingly, when PTEN-deficient thyroid cancer cells were exposed to the immunomodulatory effects of metformin (which is used for treating Type 2 diabetes), there was a more pro-inflammatory phenotype in the TME.^85^ Such a phenotype reflects a more immunogenic TME, which presents increased infiltration of immune cells together with the synthesis of cytokines and chemokines. By contrast, treatment with rapamycin (an mTOR blocker) led to reduced pro-inflammatory cytokine expression. This study provided evidence that the PI3K–AKT–mTOR network directly influences the immune response within the TME of PTEN-deficient tumours. Indeed, mTOR activation directly promotes immune-cell differentiation (reviewed in ref. ^86^). It is thus critical to confirm the multiple roles of PTEN and its related signalling pathways in the control of the inflammatory and immune responses.

### PTEN loss and immune cell infiltration

The levels of immunoreactive CD8^+^ T cells can be helpful in evaluating overall immune responses, but the immune-cell landscape of tumours that harbour *PTEN* somatic mutations is complex and highly heterogeneous, with some studies showing that PTEN deficiency is linked to a high CD8^+^ T cell density^53,87^ while other studies have shown the inverse correlation^88–90^ (Table 2).

**Table 2:** Associations between PTEN deficiency, immune cell composition and immune checkpoint expression in cancer.

Tumour type	CD8^+^	Treg	MDSC	TAM	Immune checkpoints	Sample size	Studies in murine models	Studies with human patients
Breast cancer	↑					836 patients	—	^53^
Breast cancer cell lines					↑ PD-L1	836 patients	—	^53^
Colorectal cancer					↑ PD-L1	404 patients	—	^121^
Colorectal cancer			↑			145 patients	—	^122^
Endometrial	NA					382 patients	—	^123^
Endometrial				↑		3 mice per group	—	^94^
Glioblastoma	↑ Ap.					26 patients	—	^116^
Glioblastoma				↑		66 patients	—	^22^
Glioblastoma				↑		32 patients	—	^98^
Glioma					↑ PD-L1	10 cell lines	—	^54^
Gastric and breast cancer				↑ M2		12 patients	—	^124^
HNSCC	↓	↑				5 mice per group	^88^	—
LCNC, SCLC					NA—PD-L1	189 patients	—	^125^
LSCC					↑ PD-L1	5 mice per group	^126^	—
LSCC					↓ PD-L1	102 patients	—	^127^
LUAD					↑ PD-L1	ND	^55^	—
LUAD^a^				↑ M2		13 mice	^95^	—
Melanoma	↓	↑				3 mice per group	^89^	—
Melanoma	↓					135 patients	—	^90^
Melanoma^b^	↑			↑ M2		4 mice per group	^96^	—
Melanoma cell lines					↑ PD-L1	33 patients	—	^112^
Prostate cancer				↑		70 patients	—	^74^
Prostate cancer		↑			↑ IDO1	91 patients	—	^128^
Prostate cancer	↑	↑				312 patients	—	^87^
Prostate cancer			↑			3 mice per group	^57^	—
Prostate cancer^c^			↑PMN			4 mice per group	^82^	—
Prostate cancer			↑			3 mice per group	^102^	—
Prostate cancer			↑			3 mice per group	^100^	—
Prostate cancer					NA—PD-L1	20 patients	—	^114^
Thyroid		↑	↑	↑ M2		8 mice per group	^129^	—
Uterine leiomyosarcoma					↓ PD1	1 patient	—	^107^

This is a summary of the literature of the effects of PTEN loss in various tumours based on studies of human cancer and mouse models. The arrows indicate that there is a significant association between PTEN loss, immune cell density and checkpoint expression: ↑ indicates higher density of immune cells or higher expression of immune checkpoints in PTEN-deficient tumours; ↓ indicates that there is lower cell density and expression of immune checkpoints in PTEN-deficient tumours.

*LUAD* lung adenocarcinoma, *SCC* squamous cell carcinoma, *LSCC* lung squamous cell carcinoma, *LCNC* large-cell neuroendocrine cancer, *SCLC* small-cell lung cancer, *TAM* tumour-associated macrophage, *NA* no significant association observed, *ND* not described, *HNSCC* head and neck squamous cell carcinoma. *CD8*^*+*^ CD8^+^ T cell, *Treg* regulatory T cell, *MDSC* myeloid-derived suppressor cell, *TAM* tumour-infiltrating macrophage, *PD-L1* programmed death ligand 1, *PD1* programmed death protein 1, *PMN* polymorphonuclear MDSC. *Ap.* apoptosis.

^a^*Pten*^*D5*/D5^;*Kras*^*Lox*/+^;*CCSP*^*Cre*/+^ mice.

^b^*BrafV600E*;*Pten*^−/−^ mice.

^c^*Pten*^*pc*−/−^;*Zbtb7*^*pc*−/−^ mice.

A high degree of tumour infiltration by CD8^+^ T cells is strongly linked to a better prognosis in several types of cancer.^91^ It is known that immune-cell recruitment and activation relies on tissue-specific mechanisms, which vary across different tumour types^92^ and tissues, and might even present immune-cell contents that are distinct for different lesions from a single patient.^93^ Thus it is expected that PTEN deficiency in tumour cells could elicit effects on the TME that are tumour-type specific or even tissue specific. Interestingly, several reports have shown that tumours harbouring PTEN deficiency are more likely to present with an increased M2 macrophage cell density.^22,94–96^ The presence of M2 macrophages in tumours is linked to favourable growth and invasive tumour features through the synthesis of anti-inflammatory cytokines and the inhibition of antigen presentation and cytolytic cell activation and growth.^97^

Interestingly, PTEN-deficient tumours often exhibit high densities of Treg cells in the TME (Table 2). Treg cells have an immunosuppressive effect in the TME by inactivating the priming and effector activities of CD4^+^ and CD8^+^ T cells.^59^ Moreover, several reports have confirmed the associations between the density of Treg cells and poor outcome in different types of tumour.^91^ A recently published report on glioblastoma showed that PTEN deficiency was linked to high macrophage densities.^22^ Interestingly, the authors of this study found that the density of CD8^+^ T cells increased in *PTEN* wild-type tumours after PD1 blockade therapy. Conversely, PTEN-deficient tumours did not show a significant increase in CD8^+^ T cell density after therapy. Another new study on the impact of PTEN loss in glioblastoma demonstrated increased infiltration of macrophages via the yes-associated protein 1–lysyl oxidase b1 (LOX-b1)–integrin–PYK2 axis.^98^ The authors showed that LOX expression activated specific pathways in macrophages that promoted their recruitment into the TME where they secrete the growth factor osteopontin (SPP1).

Studies with mice have shown that PTEN deficiency can increase tumour-infiltrating MDSC densities in the TME (Table 2). MDSCs comprise a heterogeneous population of myeloid progenitors and immature granulocytes, macrophages and dendritic cells. Mechanistically, MDSCs are highly immunosuppressive cells that suppress CD8^+^ T cell activity through the secretion of reactive oxygen species, peroxynitrite and nitric oxide (reviewed in ref. ^99^). MDSCs are also thought to sustain tumour growth by protecting proliferating tumour cells from senescence.^100^ Owing to the ability of MDSCs to modulate the antitumour immune response, the presence of high densities of MDSCs correlates with a poor outcome in several tumours.^91,101^ In a preclinical prostate cancer, PTEN loss results in tumours with high expression of the cytokines colony-stimulating factor 1 and Il1b, which leads to an expansion of infiltrating MDSCs.^57^ The development of castration-resistant prostate cancer—a condition in which tumours are insensitive to androgen-deprivation therapy—was found to be associated with the presence of high densities of tumour-infiltrating MDSCs and high levels of IL-23, which can activate the androgen receptor pathway in prostate tumour cells to promote cell survival and proliferation.^102^ As *PTEN* loss occurs in 40–50% of castration-resistant prostate cancers, it is critical to understand how an immunosuppressive and tumour-tolerant TME is related to increased IL-23 and infiltration of MDSCs in such advanced cancers.

Although PTEN deficiency appears to shape the TME of cancers by mediating the secretion of signalling molecules, one can speculate that these effects could also be an indirect secondary consequence of PI3K–AKT pathway dysregulation. It is known that the PI3K pathway has a role in regulating the antitumour immune response and immune-cell differentiation.^103^ Reports on PI3K blockade indicate the associations between this signalling network and macrophages, CD8^+^ T cells and Treg cells (reviewed in ref. ^104^). However, *PTEN* inactivation is sometimes reported to occur together with downstream *PIK3CA* mutations.^8^ This finding suggests that some of the somatic mutations of the *PTEN* gene are redundant but are selected for because of the loss of other regulatory functions that are independent of its role in controlling PI3K activity.

## PTEN deficiency and immune checkpoint expression

The most effective current immunotherapeutic interventions, such as PD-L1 inhibitors, are able to restore T cell activity against tumours.^105^ Unfortunately, some patients relapse after an initial response from immune-blockade therapies, suggesting that acquired tumour-specific alterations might trigger resistance to immunotherapies.^5^ As discussed above, the accumulation of somatic mutations (and thus high levels of neoantigens) and pathway dysregulation of tumours might lead to an altered immune cell composition in the TME that strongly influences therapy responses.^105^

As a result of innate immune resistance, the constitutive activation of oncogenic pathways can promote the synthesis of PD-L1 in tumour cells independently of the immune-cell state in the TME. For instance, in murine models of lung cancer, activation of the AKT–mTOR pathway promoted the expression of PD-L1.^55^ Functional studies with lung cancer cell lines also demonstrated that tumour-infiltrating macrophages might promote PD-L1 synthesis by secreting IFN-γ in a JAK–STAT3-dependent manner.^106^ Interestingly, PTEN-deficient melanomas and sarcomas express high levels of VEGFA and STAT3, while also showing resistance to ICIs.^30,107^ If PTEN loss occurs, the upregulation of angiogenesis, as mediated by VEGFA and STAT3, may also influence the trafficking of immune cells in the TME.^108,109^ It is known that the tumour blood vessels can be modulated by both inflammatory triggers and the types of infiltrating immune cell subsets in the TME. To infiltrate into the tumour and its TME, immune cells must enter the tumour vasculature, adhere to the endothelium and migrate across the blood vessel wall. For these reasons, there is interest in combining anti-angiogenic agents with ICIs to potentially improve immunotherapy responses (reviewed in ref. ^110^).

Several studies in breast cancer, colorectal cancer and glioma have shown that cancer cell intrinsic PD-L1 expression increases as a result of loss of PTEN. This finding suggests that the context of pre-existing tumour immune states could be informative for more precise use of immunotherapy (Table 2). Through in silico analyses of The Cancer Genome Atlas sarcoma specimens, it was shown that the presence of an oncogenic mutation and a heterozygous deletion in the remaining *PTEN* allele led to a lower expression of CD8^+^ T cell markers (granzyme A [*GZMA*], perforin 1 [*PRF1*], and cluster of differentiation 8A [*CD8A*]) and *PD1* and interferon γ (*IFNG*) genes.^107^ This observation indicates that complete inactivation of *PTEN* is associated with a reduced CD8^+^ T cell density and activation within the sarcoma TME. In murine lung cancer models, activation of the PI3K–AKT–mTOR cascade resulting from PTEN deficiency was associated with the overexpression of PD-L1.^111^ This finding indicates that PTEN loss and consequent overactivation of the PI3K–AKT–mTOR induces PD-L1 expression. The authors also showed that tumour growth was inhibited, the number of tumour-infiltrating T cells was increased and the density of Treg cells decreased with the combined treatment of mTOR inhibition and anti-PD1 agents.

In PTEN-deficient breast cancer cell lines, Mittendorf and colleagues^53^ observed overexpression of PD1 and PD-L1, which was downregulated after PI3K pathway inhibition. Moreover, PD-L1 overexpression induced by PTEN loss in tumour cells was associated with low T cell proliferation rates.^53^ Similarly, the absence of PTEN expression in melanoma cell lines was associated with the upregulation of PD-L1 expression.^112^ In gliomas, PTEN loss was significantly associated with PD-L1 overexpression.^54^ *PTEN* mutations have been linked to anti-PD1 therapy resistance in glioblastomas.^22^ Indeed, PTEN-intact glioblastomas showed a high density of CD3^+^CD8^+^ cells after treatment with PD1 inhibitors, but *PTEN*-deficient tumours did not exhibit this effect.

In a murine model of castration-resistant prostate cancer, the combination of deletions of *Pten*, transformation-related protein 53 (*Trp53*) and *Smad4* led to resistance to anti-PD1 and anti-CTLA4 antibodies.^113^ Interestingly, PTEN deficiency alone was not associated with PD-L1 expression in prostate cancer. Indeed, PD-L1 expression in radical prostatectomy-derived prostate tumours is rare.^114^ Collectively, these observations demonstrate that knowledge of the status of PTEN might be useful in deciding which patients and which specific tumour types might benefit from current immunotherapeutic approaches.

## Clinical trials investigating the influence of PTEN on the response to immunotherapy

As PTEN functions determine how tumour cells react to the immune response in the TME, this gene might be a useful biomarker for determining how patients will respond to therapy. A Phase 2 trial showed that patients with PTEN-deficient castration-resistant prostate cancer had improved radiographic progression-free survival when treated with a combination of the hormone therapy drug abiraterone and an AKT blocker.^115^ Although PTEN biomarker testing has been a consideration for standard therapeutic responses, only a limited number of clinical trials plan to determine PTEN status in relation to the clinical response of tumours with ICIs (more details in Table 3). This suggests that PTEN might have been overlooked in trial designs, despite the compelling associations between this pathway and immunotherapy response. However, several downstream markers of the PTEN signalling pathway, such as AKT and PI3K, are being investigated in conjunction with checkpoint blockade therapies.

**Table 3:** Clinical trials investigating immune response biomarkers and downstream effectors of PTEN–PI3K–AKT–mTOR pathway using checkpoint blockade therapies.

PTEN-associated mechanism	Tumour type	Drug	Study details	Trial number
PTEN loss and phospho-AKT	Non-small cell lung carcinoma (NSCLC)	AZD6244 (KRAS mutant patients) Erlotinib (wild-type KRAS patients) AZD6244+Erlotinib	Phospho-ERK (p-ERK), phospho-protein kinase B (p-AKT) and PTEN expression will be determined. PD1 expression will be investigated in Tregs and CD8^+^ T cells	NCT01229150
PTEN loss and AKT	Advanced or metastatic solid tumour malignancies	AZD5363 (AKT blockade) Durvalumab (anti-PD1)	Investigate the links between mutations in Akt/PIK3CA/PTEN pathway and response to AZD5363+Olaparib+Durvalumab. To understand the role of Tregs in improving response to Durvalumab. Determine the role of AZD5363 as an immunomodulator	NCT03772561
Phospho-AKT	Stage I–IV oral and oropharyngeal squamous cell carcinoma	Metformin hydrochloride/pioglitazone hydrochloride extended-release tablet	PD1 and PD-L1 expression will be compared between patients before and after treatment. IHC will be performed with (p)AKT, pAMPK, pS6 and tumour-infiltrating immune cells (CD8, IFNγ, Treg and CD68)	NCT02917629
Phospho-AKT	NSCLC Squamous cell adenocarcinoma	AZD5363 (AKT inhibitor) Durvalumab (anti-PD-L1) Other drugs (ZD4547; Vistusertib; Palbociclib; Crizotinib; Selumetinib; Docetaxel; Osimertinib; Sitravatinib)	Multi-drug and genetic testing in a multi-arm Phase 2 trial. No genomic or expression tests.	NCT02664935
Phospho-AKT	Metastatic breast cancer	MEDI4736 (anti-PD-L1) AZD5363 (AKT inhibitor) Other drugs	DNA will be investigated by NGS and microarray	NCT02299999
Phospho-AKT	NSCLC	MEDI4736 (anti-PD-L1) AZD5363 (AKT inhibitor)	DNA will be investigated by NGS and microarray.	NCT02117167
PI3K inhibition	Unresectable or metastatic microsatellite-stable solid tumour along with microsatellite-stable colon cancer Colon cancer	Copanlisib (PI3K inhibitor) Nivolumab (anti-PD1)	Phase 1/2 study of PI3K inhibition (Copanlisib) and anti-PD1 (Nivolumab) in refractory mismatch-repair proficient (MSS) colorectal tumours. No genomic or expression tests	NCT03711058
PI3K inhibition	Classical Hodgkin lymphoma	Tenalisib Pembrolizumab	Phase 1/2 study to investigate the safety and efficacy of RP6530 (PI3Kδ/γ dual inhibitor) in combination with an anti-PD1 therapy (pembrolizumab). No genomic or expression tests	NCT03471351
PI3K inhibition	Metastatic NSCLC	Abemaciclib	NGS for 245 genes, NanoString nCounter including immune signature and IHC with PD-L1 in patients treated with PI3K inhibitor and PD1/PD-L1 inhibitors	NCT03356587
PI3K inhibition	Advanced solid tumours	Itacitinib Epacadostat INCB050465	JAK inhibitor with JAK1 selectivity (Itacitinib) in combination with an IDO1 inhibitor (epacadostat; INCB024360; Group A) and Itacitinib in combination with a PI3Kδ inhibitor (INCB050465; Group B)	NCT02559492

These clinical trials and their associated biomarker studies may provide more information of the impact of PTEN/PI3K on responses to various drugs and checkpoint inhibitors in different solid tumours.

*IHC* immunohistochemistry, *NGS* next-generation sequencing, *pAMPK* phosphorylated AMP-activated protein kinase, *Treg* regulatory T cell.

Interestingly, inhibition of components of the PI3K–AKT–mTOR network in PTEN-deficient primary cultures of gliomas led to a decrease in T cell death.^116^ The effects of restoration of PI3K control suggests that PTEN loss in tumours may be enhancing the adaptive immune response against cancer cells.^117^ Concordantly, ablation of the PI3K pathway led to a marked enhancement of the antitumour functions of Toll-like receptor ligands in different murine models of cancer.^118^ The outcome of several ongoing trials investigating genomic alterations and potential links between PTEN loss and response to ICIs (Table 3) could be very informative. The trials (NCT02299999, NCT02117167 and NCT03772561) will involve transcriptome analysis, so it will be possible to determine whether genomic changes such as PTEN loss, combined with other mutations, are involved in the differential response to immunotherapy. In this manner, determining PTEN deficiency in tumours might be useful for future biomarker-guided combination drug trials.

## Concluding remarks

Various lines of evidence suggest that loss of PTEN has a crucial role in the development of an immunosuppressive cancer phenotype for some tumours. As restoring the function of PTEN is presently not feasible,^119^ the most obvious way to counter the effects of PTEN loss would be to inhibit PI3K signalling. However, as PTEN and PTEN-L appear to mediate immune responses independently of PI3K, future therapeutic approaches should target other downstream pathways and signalling molecules that directly control TME immune responses. For instance, blockade of the JAK–STAT3 pathway in *PTEN*-null prostate cancers induced the TME to become more immunogenic and to be infiltrated by increased numbers of T cells.^46^ In this way, tumours could be primed to respond to anti-PD1 or anti-PD-L1 therapy as the immune cells in the TME can be induced to better recognise tumour cells. Other approaches, such as anti-angiogenic therapies, might also benefit patients before exposure to immunotherapeutics.^11^ Interestingly, PTEN-deficient glioblastomas overexpressed the CD44 cell-surface adhesion receptor and had a more compact tumour-cell phenotype that could exclude vascularisation and immune cells from the TME than wild-type glioblastomas,^22^ rendering them less likely to respond to ICI. More studies are required to determine the mechanistic links between PTEN, angiogenesis and the response to anti-PD1/PD-L1. As many patients experience relapse after immunotherapy, it is critical to characterise new tumour-specific genomic biomarkers and the molecular signatures of response that will be informative for immune-based treatments.

## Data Availability

Not applicable.

## References

[1] Wang P, Zhang H, Yang J, Li Z, Wang Y, Leng X (2020). Mu–KRAS attenuates Hippo signaling pathway through PKCι to sustain the growth of pancreatic cancer. J. Cell. Physiol..

[2] Cantley LC, Neel BG (1999). New insights into tumor suppression: PTEN suppresses tumor formation by restraining the phosphoinositide 3-kinase/AKT pathway. Proc. Natl Acad. Sci..

[3] Adams JM, Harris AW, Pinkert CA, Corcoran LM, Alexander WS, Cory S (1985). The c-myc oncogene driven by immunoglobulin enhancers induces lymphoid malignancy in transgenic mice. Nature.

[4] Fisher GH (2001). Induction and apoptotic regression of lung adenocarcinomas by regulation of a K-Ras transgene in the presence and absence of tumor suppressor genes. Genes Dev..

[5] Wellenstein MD, de Visser KE (2018). Cancer-cell-intrinsic mechanisms shaping the tumor immune landscape. Immunity.

[6] Rooney MSS, Shukla SAA, Wu CJJ, Getz G, Hacohen N (2015). Molecular and genetic properties of tumors associated with local immune cytolytic activity. Cell.

[7] Lee Y-R, Chen M, Pandolfi PP (2018). The functions and regulation of the PTEN tumour suppressor: new modes and prospects. Nat. Rev. Mol. Cell Biol..

[8] Millis SZ, Ikeda S, Reddy S, Gatalica Z, Kurzrock R (2016). Landscape of phosphatidylinositol-3-kinase pathway alterations across 19 784 diverse solid tumors. JAMA Oncol.

[9] Yehia L, Ngeow J, Eng C (2019). PTEN-opathies: from biological insights to evidence-based precision medicine. J. Clin. Invest..

[10] Nowak DG, Cho H, Herzka T, Watrud K, DeMarco DV, Wang VMY (2015). MYC drives Pten/Trp53-deficient proliferation and metastasis due to IL6 secretion and AKT suppression via PHLPP2. Cancer Discov..

[11] Cheng F, Eng C (2019). PTEN mutations trigger resistance to immunotherapy. Trends Mol. Med..

[12] Chen L, Guo D (2017). The functions of tumor suppressor PTEN in innate and adaptive immunity. Cell Mol. Immunol..

[13] Li S, Zhu M, Pan R, Fang T, Cao Y-Y, Chen S (2015). The tumor suppressor PTEN has a critical role in antiviral innate immunity. Nat. Immunol..

[14] Cao Y, Wang H, Yang L, Zhang Z, Li CC-M, Yuan X (2018). PTEN-L promotes type I interferon responses and antiviral immunity. Cell Mol. Immunol..

[15] Horton BL, Williams JB, Cabanov A, Spranger S, Gajewski TF (2018). Intratumoral CD8 + T-cell apoptosis is a major component of T-cell dysfunction and impedes antitumor immunity. Cancer Immunol. Res..

[16] Kryczek I, Lange A, Mottram P, Alvarez X, Cheng P, Hogan M (2005). CXCL12 and vascular endothelial growth factor synergistically induce neoangiogenesis in human ovarian cancers. Cancer Res..

[17] Allen F, Bobanga ID, Rauhe P, Barkauskas D, Teich N, Tong C (2018). CCL3 augments tumor rejection and enhances CD8 + T cell infiltration through NK and CD103 + dendritic cell recruitment via IFNγ. Oncoimmunology.

[18] Muthuswamy R, Corman JM, Dahl K, Chatta GS, Kalinski P (2016). Functional reprogramming of human prostate cancer to promote local attraction of effector CD8 ^+^ T cells. Prostate.

[19] Segal NH, Parsons DW, Peggs KS, Velculescu V, Kinzler KW, Vogelstein B (2008). Epitope landscape in breast and colorectal cancer. Cancer Res..

[20] Tanaka A, Sakaguchi S (2017). Regulatory T cells in cancer immunotherapy. Cell Res..

[21] Tumeh PC, Harview CL, Yearley JH, Shintaku IP, Taylor EJM, Robert L (2014). PD-1 blockade induces responses by inhibiting adaptive immune resistance. Nature.

[22] Zhao J, Chen AX, Gartrell RD, Silverman AM, Aparicio L, Chu T (2019). Immune and genomic correlates of response to anti-PD-1 immunotherapy in glioblastoma. Nat. Med..

[23] Koyama S, Akbay EA, Li YY, Herter-Sprie GS, Buczkowski KA, Richards WG (2016). Adaptive resistance to therapeutic PD-1 blockade is associated with upregulation of alternative immune checkpoints. Nat. Commun..

[24] Deng R, Fan F, Yi H, Liu F, He G, Sun H (2018). PD-1 blockade potentially enhances adoptive cytotoxic T cell potency in a human acute myeloid leukaemia animal model. Hematology.

[25] Della Corte CM, Barra G, Ciaramella V, Di Liello R, Vicidomini G, Zappavigna S (2019). Antitumor activity of dual blockade of PD-L1 and MEK in NSCLC patients derived three-dimensional spheroid cultures. J. Exp. Clin. Cancer Res..

[26] Borcoman E, Kanjanapan Y, Champiat S, Kato S, Servois V, Kurzrock R (2019). Novel patterns of response under immunotherapy. Ann. Oncol..

[27] Goodman AM, Kato S, Bazhenova L, Patel SP, Frampton GM, Miller V (2017). Tumor mutational burden as an independent predictor of response to immunotherapy in diverse cancers. Mol. Cancer Ther..

[28] Quigley D, Silwal-Pandit L, Dannenfelser R, Langerød A, Vollan HKM, Vaske C (2015). Lymphocyte invasion in IC10/basal-like breast tumors is associated with wild-type TP53. Mol. Cancer Res.

[29] Wang Q, Hu B, Hu X, Kim H, Squatrito M, Scarpace L (2017). Tumor evolution of glioma-intrinsic gene expression subtypes associates with immunological changes in the microenvironment. Cancer Cell.

[30] Peng W, Chen JQ, Liu C, Malu S, Creasy C, Tetzlaff MT (2016). Loss of PTEN promotes resistance to T cell-mediated immunotherapy. Cancer Discov..

[31] Rizvi NA, Hellmann MD, Snyder A, Kvistborg P, Makarov V, Havel JJ (2015). Mutational landscape determines sensitivity to PD-1 blockade in non–small cell lung cancer. Science.

[32] Thorsson V, Gibbs DL, Brown SD, Wolf D, Bortone DS, Ou Yang TH (2018). The immune landscape of cancer. Immunity.

[33] Jamaspishvili T, Berman DM, Ross AE, Scher HI, De Marzo AM, Squire JA (2018). Clinical implications of PTEN loss in prostate cancer. Nat. Rev. Urol..

[34] Lee J-O, Yang H, Georgescu M-M, Di Cristofano A, Maehama T, Shi Y (1999). Crystal structure of the PTEN tumor suppressor. Cell.

[35] Steck PA, Pershouse MA, Jasser SA, Yung WKA, Lin H, Ligon AH (1997). Identification of a candidate tumour suppressor gene, MMAC1, at chromosome 10q23.3 that is mutated in multiple advanced cancers. Nat. Genet..

[36] Maehama T, Dixon JE (1998). The tumor suppressor, PTEN/MMAC1, dephosphorylates the lipid second messenger, phosphatidylinositol 3,4,5-trisphosphate. J. Biol. Chem..

[37] Lee JO, Yang H, Georgescu MM, Di Cristofano A, Maehama T, Shi Y (1999). Crystal structure of the PTEN tumor suppressor: Implications for its phosphoinositide phosphatase activity and membrane association. Cell.

[38] Bassi C, Ho J, Srikumar T, Dowling RJO, Gorrini C, Miller SJ (2013). Nuclear PTEN controls DNA repair and sensitivity to genotoxic stress. Science.

[39] Shen WH, Balajee AS, Wang J, Wu H, Eng C, Pandolfi PP (2007). Essential role for nuclear PTEN in maintaining chromosomal integrity. Cell.

[40] Ma J, Benitez JA, Li J, Miki S, Ponte de Albuquerque C, Galatro T (2019). Inhibition of nuclear PTEN tyrosine phosphorylation enhances glioma radiation sensitivity through attenuated DNA repair. Cancer Cell.

[41] Zhao D, Lu X, Wang G, Lan Z, Liao W, Li J (2017). Synthetic essentiality of chromatin remodelling factor CHD1 in PTEN-deficient cancer. Nature.

[42] Collado M, Blasco MA, Serrano M (2007). Cellular senescence in cancer and aging. Cell.

[43] Jung SH, Hwang HJ, Kang D, Park HA, Lee HC, Jeong D (2019). mTOR kinase leads to PTEN-loss-induced cellular senescence by phosphorylating p53. Oncogene.

[44] Chen Z, Trotman LC, Shaffer D, Lin H-K, Dotan ZA, Niki M (2005). Crucial role of p53-dependent cellular senescence in suppression of Pten-deficient tumorigenesis. Nature.

[45] Song MS, Carracedo A, Salmena L, Song SJ, Egia A, Malumbres M (2011). Nuclear PTEN regulates the APC-CDH1 tumor-suppressive complex in a phosphatase-independent manner. Cell.

[46] Toso A, Revandkar A, DiMitri D, Guccini I, Proietti M, Sarti M (2014). Enhancing chemotherapy efficacy in pten-deficient prostate tumors by activating the senescence-associated antitumor immunity. Cell Rep..

[47] Vidotto T, Tiezzi DG, Squire JA (2018). Distinct subtypes of genomic PTEN deletion size influence the landscape of aneuploidy and outcome in prostate cancer. Mol. Cytogenet..

[48] Williams JL, Greer PA, Squire JA (2014). Recurrent copy number alterations in prostate cancer: an in silico meta-analysis of publicly available genomic data. Cancer Genet..

[49] Taylor AM, Shih J, Ha G, Gao GF, Zhang X, Berger AC (2018). Genomic and functional approaches to understanding cancer aneuploidy. Cancer Cell.

[50] Davoli T, Uno H, Wooten EC, Elledge SJ (2017). Tumor aneuploidy correlates with markers of immune evasion and with reduced response to immunotherapy. Science.

[51] Turajlic S, Litchfield K, Xu H, Rosenthal R, McGranahan N, Reading JL (2017). Insertion-and-deletion-derived tumour-specific neoantigens and the immunogenic phenotype: a pan-cancer analysis. Lancet Oncol..

[52] Dudley JC, Lin M-T, Le DT, Eshleman JR (2016). Microsatellite instability as a biomarker for PD-1 blockade. Clin. Cancer Res..

[53] Mittendorf EA, Philips AV, Meric-Bernstam F, Qiao N, Wu Y, Harrington S (2014). PD-L1 expression in triple-negative breast cancer. Cancer Immunol. Res..

[54] Parsa AT, Waldron JS, Panner A, Crane CA, Parney IF, Barry JJ (2007). Loss of tumor suppressor PTEN function increases B7-H1 expression and immunoresistance in glioma. Nat. Med..

[55] Lastwika KJ, Wilson W, Li QK, Norris J, Xu H, Ghazarian SR (2016). Control of PD-L1 expression by oncogenic activation of the AKT–mTOR pathway in non–small cell lung cancer. Cancer Res.

[56] Berghoff AS, Kiesel B, Widhalm G, Rajky O, Ricken G, Wohrer A (2015). Programmed death ligand 1 expression and tumor-infiltrating lymphocytes in glioblastoma. Neuro Oncol..

[57] Garcia AJ, Ruscetti M, Arenzana TL, Tran LM, Bianci-Frias D, Sybert E (2014). Pten null prostate epithelium promotes localized myeloid-derived suppressor cell expansion and immune suppression during tumor initiation and progression. Mol. Cell Biol..

[58] Miething C, Scuoppo C, Bosbach B, Appelmann I, Nakitandwe J, Ma J (2014). PTEN action in leukaemia dictated by the tissue microenvironment. Nature.

[59] Colombo MP, Piconese S (2007). Regulatory T-cell inhibition versus depletion: the right choice in cancer immunotherapy. Nat. Rev. Cancer.

[60] Ivashkiv LB, Donlin LT (2013). Regulation of type I interferon responses. Nat. Rev. Immunol..

[61] Shen H, Lentsch AB (2004). Progressive dysregulation of transcription factors NF-κB and STAT1 in prostate cancer cells causes proangiogenic production of CXC chemokines. Am. J. Physiol. Physiol..

[62] Fuertes MB, Woo SR, Burnett B, Fu YX, Gajewski TF (2013). Type I interferon response and innate immune sensing of cancer. Trends Immunol..

[63] Parker BS, Rautela J, Hertzog PJ (2016). Antitumour actions of interferons: implications for cancer therapy. Nat. Rev. Cancer.

[64] Bakhoum SF, Cantley LC (2018). The multifaceted role of chromosomal instability in cancer and its microenvironment. Cell.

[65] Mouw KW, Goldberg MS, Konstantinopoulos PA, D’Andrea AD (2017). DNA damage and repair biomarkers of immunotherapy response. Cancer Discov..

[66] Wang H, Yu Q, Wang L, Li Y, Xie M, Lu Y (2018). Expression of PTEN‑long nephritis and its effect on renal inflammation. Exp. Ther. Med..

[67] Bazzichetto C, Conciatori F, Pallocca M, Falcone I, Fanciulli M, Cognetti F (2019). PTEN as a prognostic/predictive biomarker in cancer: an unfulfilled promise?. Cancers (Basel).

[68] Newton RH, Turka LA (2012). Regulation of T cell homeostasis and responses by Pten. Front. Immunol..

[69] Shrestha S, Yang K, Guy C, Vogel P, Neale G, Chi H (2015). Treg cells require the phosphatase PTEN to restrain TH1 and TFH cell responses. Nat. Immunol..

[70] Suzuki A, Kaisho T, Ohishi M, Tsukio-Yamaguchi M, Tsubata T, Koni PA (2003). Critical roles of Pten in B cell homeostasis and immunoglobulin class switch recombination. J. Exp. Med..

[71] Sahin E, Haubenwallner S, Kuttke M, Kollmann I, Halfmann A, Dohnal AB (2014). Macrophage PTEN regulates expression and secretion of arginase I modulating innate and adaptive immune responses. J. Immunol..

[72] Di Cristofano A, Kotsi P, Peng YF, Cordon-Cardo C, Elkon KB, Pandolfi PP (1999). Impaired Fas response and autoimmunity in Pten+/- mice. Science.

[73] Grivennikov SI, Greten FR, Karin M (2010). Immunity, inflammation, and cancer. Cell.

[74] Armstrong CWD, Maxwell PJ, Ong CW, Redmond KM, Mccann C, Neisen J (2016). PTEN deficiency promotes macrophage infiltration and hypersensitivity of prostate cancer to IAP antagonist/radiation combination therapy. Oncotarget.

[75] Chelouche-Lev D, Miller CP, Tellez C, Ruiz M, Bar-Eli M, Price JE (2004). Different signalling pathways regulate VEGF and IL-8 expression in breast cancer: implications for therapy. Eur. J. Cancer.

[76] Ying H, Elpek KG, Vinjamoori A, Zimmerman SM, Chu GC, Yan H (2011). PTEN is a major tumor suppressor in pancreatic ductal adenocarcinoma and regulates an NF-κB-cytokine network. Cancer Discov..

[77] Loveridge CJ, Mui EJ, Patel R, Tan EH, Ahmad I, Welsh M (2017). Increased T-cell infiltration elicited by Erk5 deletion in a Pten-deficient mouse model of prostate carcinogenesis. Cancer Res..

[78] Phillips RJ, Mestas J, Gharaee-Kermani M, Burdick MD, Sica A, Belperio JA (2005). Epidermal growth factor and hypoxia-induced expression of CXC chemokine receptor 4 on non-small cell lung cancer cells is regulated by the phosphatidylinositol 3-kinase/PTEN/AKT/mammalian target of rapamycin signaling pathway and activation of hypoxia inducible factor-1α. J. Biol. Chem..

[79] Trimboli AJ, Cantemir-Stone CZ, Li F, Wallace JA, Merchant A, Creasap N (2009). Pten in stromal fibroblasts suppresses mammary epithelial tumours. Nature.

[80] Conley-LaComb M, Saliganan A, Kandagatla P, Chen YQ, Cher ML, Chinni SR (2013). PTEN loss mediated Akt activation promotes prostate tumor growth and metastasis via CXCL12/CXCR4 signaling. Mol. Cancer.

[81] Maxwell PJ, Neisen J, Messenger J, Waugh DJJ (2014). Tumor-derived CXCL8 signaling augments stroma-derived CCL2-promoted proliferation and CXCL12-mediated invasion of PTEN-deficient prostate cancer cells. Oncotarget.

[82] Bezzi M, Seitzer N, Ishikawa T, Reschke M, Chen M, Wang G (2018). Diverse genetic-driven immune landscapes dictate tumor progression through distinct mechanisms. Nat. Med..

[83] Hollander MC, Blumenthal GM, Dennis PA (2011). PTEN loss in the continuum of common cancers, rare syndromes and mouse models. Nat. Rev. Cancer.

[84] Nieuwenhuis MH, Kets CM, Murphy-Ryan M, Yntema HG, Evans DG, Colas C (2014). Cancer risk and genotype-phenotype correlations in PTEN hamartoma tumor syndrome. Fam. Cancer.

[85] Sloot YJE, Rabold K, Netea MG, Smit JWA, Hoogerbrugge N, Netea-Maier RT (2019). Effect of PTEN inactivating germline mutations on innate immune cell function and thyroid cancer-induced macrophages in patients with PTEN hamartoma tumor syndrome. Oncogene.

[86] Conciatori F, Bazzichetto C, Falcone I, Pilotto S, Bria E, Cognetti F (2018). Role of mTOR signaling in tumor microenvironment: an overview. Int. J. Mol. Sci..

[87] Kaur HB, Guedes LB, Lu J, Maldonado L, Reitz L, Barber JR (2018). Association of tumor-infiltrating T-cell density with molecular subtype, racial ancestry and clinical outcomes in prostate cancer. Mod. Pathol..

[88] Bian Y, Hall B, Sun Z-J, Molinolo A, Chen W, Gutkind JS (2012). Loss of TGF-β signaling and PTEN promotes head and neck squamous cell carcinoma through cellular senescence evasion and cancer-related inflammation. Oncogene.

[89] Shabaneh TB, Molodtsov AK, Steinberg SM, Zhang P, Torres GM, Mohamed GA (2018). Oncogenic BRAFV600E governs regulatory T-cell recruitment during melanoma tumorigenesis. Cancer Res.

[90] Peng W, Chen JQ, Liu C, Malu S, Creasy C, Tetzlaff MT (2016). Loss of PTEN promotes resistance to T cell–mediated immunotherapy. Cancer Discov..

[91] Fridman WH, Zitvogel L, Sautès-Fridman C, Kroemer G, Sautès–Fridman C, Kroemer G (2017). The immune contexture in cancer prognosis and treatment. Nat. Rev. Clin. Oncol..

[92] Gentles AJ, Newman AM, Liu CL, Bratman SV, Feng W, Kim D (2015). The prognostic landscape of genes and infiltrating immune cells across human cancers. Nat. Med..

[93] Jiménez-Sánchez A, Memon D, Pourpe S, Veeraraghavan H, Li Y, Vargas HA (2017). Heterogeneous tumor-immune microenvironments among differentially growing metastases in an ovarian cancer patient. Cell.

[94] Liang X, Daikoku T, Terakawa J, Ogawa Y, Joshi AR, Ellenson LH (2018). The uterine epithelial loss of Pten is inefficient to induce endometrial cancer with intact stromal Pten. PLoS Genet..

[95] Iwanaga K, Yang Y, Raso MG, Ma L, Hanna AE, Thilaganathan N (2008). Pten inactivation accelerates oncogenic K-ras-initiated tumorigenesis in a mouse model of lung cancer. Cancer Res..

[96] Homet Moreno B, Zaretsky JM, Garcia-Diaz A, Tsoi J, Parisi G, Robert L (2016). Response to programmed cell death-1 blockade in a murine melanoma syngeneic model requires costimulation, CD4, and CD8 T cells. Cancer Immunol. Res..

[97] Almatroodi SA, McDonald CF, Darby IA, Pouniotis DS (2016). Characterization of M1/M2 tumour-associated macrophages (TAMs) and Th1/Th2 cytokine profiles in patients with NSCLC. Cancer Microenviron..

[98] Chen P, Zhao D, Li J, Liang X, Li J, Chang A (2019). Symbiotic macrophage-glioma cell interactions reveal synthetic lethality in PTEN-null glioma. Cancer Cell.

[99] Gabrilovich DI, Nagaraj S (2009). Myeloid-derived suppressor cells as regulators of the immune system. Nat. Rev. Immunol..

[100] Di Mitri D, Toso A, Chen JJ, Sarti M, Pinton S, Jost TR (2014). Tumour-infiltrating Gr-1+ myeloid cells antagonize senescence in cancer. Nature.

[101] Limagne E, Euvrard R, Thibaudin M, Rébé C, Derangère V, Chevriaux A (2016). Accumulation of MDSC and Th17 cells in patients with metastatic colorectal cancer predicts the efficacy of a FOLFOX–bevacizumab drug treatment regimen. Cancer Res..

[102] Calcinotto A, Spataro C, Zagato E, Di Mitri D, Gil V, Crespo M (2018). IL-23 secreted by myeloid cells drives castration-resistant prostate cancer. Nature.

[103] Koyasu S (2003). The role of PI3K in immune cells. Nat. Immunol..

[104] Okkenhaug K, Graupera M, Vanhaesebroeck B (2016). Targeting PI3K in cancer: impact on tumor cells, their protective stroma, angiogenesis, and immunotherapy. Cancer Discov..

[105] Pitt JM, Vétizou M, Daillère R, Roberti MP, Yamazaki T, Routy B (2016). Resistance mechanisms to immune-checkpoint blockade in cancer: tumor-intrinsic and -extrinsic factors. Immunity.

[106] Zhang X, Zeng Y, Qu Q, Zhu J, Liu Z, Ning W (2017). PD-L1 induced by IFN-γ from tumor-associated macrophages via the JAK/STAT3 and PI3K/AKT signaling pathways promoted progression of lung cancer. Int. J. Clin. Oncol..

[107] George S, Miao D, Demetri GD, Adeegbe D, Rodig SJ, Shukla S (2017). Loss of PTEN is associated with resistance to anti-PD-1 checkpoint blockade therapy in metastatic uterine leiomyosarcoma. Immunity.

[108] Jin H, Su J, Garmy-Susini B, Kleeman J, Varner J (2006). Integrin α4β1 promotes monocyte trafficking and angiogenesis in tumors. Cancer Res..

[109] Roland CL, Dineen SP, Lynn KD, Sullivan LA, Dellinger MT, Sadegh L (2009). Inhibition of vascular endothelial growth factor reduces angiogenesis and modulates immune cell infiltration of orthotopic breast cancer xenografts. Mol. Cancer Ther..

[110] Fukumura D, Kloepper J, Amoozgar Z, Duda DG, Jain RK (2018). Enhancing cancer immunotherapy using antiangiogenics: opportunities and challenges. Nat. Rev. Clin. Oncol..

[111] Lastwika KJ, Wilson W, Li QK, Norris J, Xu H, Ghazarian SR (2016). Control of PD-L1 expression by oncogenic activation of the AKT-mTOR pathway in non-small cell lung cancer. Cancer Res..

[112] Dong Y, Richards JA, Gupta R, Aung PP, Emley A, Kluger Y (2014). PTEN functions as a melanoma tumor suppressor by promoting host immune response. Oncogene.

[113] Feng S, Cheng X, Zhang L, Lu X, Chaudhary S, Teng R (2018). Myeloid-derived suppressor cells inhibit T cell activation through nitrating LCK in mouse cancers. Proc. Natl Acad. Sci. USA.

[114] Martin AM, Nirschl TR, Nirschl CJ, Francica BJ, Kochel CM, van Bokhoven A (2015). Paucity of PD-L1 expression in prostate cancer: innate and adaptive immune resistance. Prostate Cancer Prostatic Dis..

[115] de Bono JS, De Giorgi U, Rodrigues DN, Massard C, Bracarda S, Font A (2019). Randomized phase II study evaluating Akt blockade with ipatasertib, in combination with abiraterone, in patients with metastatic prostate cancer with and without PTEN loss. Clin. Cancer Res..

[116] Waldron JS, Yang I, Han S, Tihan T, Sughrue ME, Mills SA (2010). Implications for immunotherapy of tumor-mediated T-cell apoptosis associated with loss of the tumor suppressor PTEN in glioblastoma. J. Clin. Neurosci..

[117] Lu B, Finn OJ (2008). T-cell death and cancer immune tolerance. Cell Death Differ..

[118] Marshall NA, Galvin KC, Corcoran A-MB, Boon L, Higgs R, Mills KHG (2012). Immunotherapy with PI3K inhibitor and toll-like receptor agonist induces IFN- +IL-17+ polyfunctional T cells that mediate rejection of murine tumors. Cancer Res..

[119] McLoughlin NM, Mueller C, Grossmann TN (2018). The therapeutic potential of PTEN modulation: targeting strategies from gene to protein. Cell Chem. Biol..

[120] Lotan TL, Wei W, Ludkovski O, Morais CL, Guedes LB, Jamaspishvili T (2016). Analytic validation of a clinical-grade PTEN immunohistochemistry assay in prostate cancer by comparison with PTEN FISH. Mod. Pathol..

[121] Song M, Chen D, Lu B, Wang C, Zhang J, Huang L (2013). PTEN loss increases PD-L1 protein expression and affects the correlation between PD-L1 expression and clinical parameters in colorectal cancer. PLoS ONE.

[122] Yang R, Cai T, Wu X, Liu Y, He J, Zhang X (2018). Tumour YAP1 and PTEN expression correlates with tumour-associated myeloid suppressor cell expansion and reduced survival in colorectal cancer. Immunology.

[123] Crumley S, Kurnit K, Hudgens C, Fellman B, Tetzlaff MT, Broaddus R (2019). Identification of a subset of microsatellite-stable endometrial carcinoma with high PD-L1 and CD8+ lymphocytes. Mod. Pathol..

[124] Li N, Qin J, Lan L, Zhang H, Liu F, Wu Z (2015). PTEN inhibits macrophage polarization from M1 to M2 through CCL2 and VEGF-A reduction and NHERF-1 synergism. Cancer Biol. Ther..

[125] Kim HS, Lee JH, Nam SJ, Ock CY, Moon JW, Yoo CW (2018). Association of PD-L1 expression with tumor-infiltrating immune cells and mutation burden in high-grade neuroendocrine carcinoma of the lung. J. Thorac. Oncol..

[126] Xu C, Fillmore CM, Koyama S, Wu H, Zhao Y, Chen Z (2014). Loss of Lkb1 and Pten leads to lung squamous cell carcinoma with elevated PD–L1 expression. Cancer Cell.

[127] Hlaing AM, Furusato B, Udo E, Kitamura Y, Souda M, Masutani M (2018). Expression of phosphatase and tensin homolog and programmed cell death ligand 1 in adenosquamous carcinoma of the lung. Biochem. Biophys. Res. Commun..

[128] Vidotto T, Saggioro FP, Jamaspishvili T, Chesca DL, Picanço de Albuquerque CG, Reis RB (2019). PTEN–deficient prostate cancer is associated with an immunosuppressive tumor microenvironment mediated by increased expression of IDO1 and infiltrating FoxP3+ T regulatory cells. Prostate.

[129] Jolly LA, Massoll N, Franco AT (2016). Immune suppression mediated by myeloid and lymphoid derived immune cells in the tumor microenvironment facilitates progression of thyroid cancers driven by Hras^G12V^ and Pten loss. J. Clin. Cell Immunol..

